# Self-Sacrificial Salt Templating: Simple Auxiliary Control over the Nanoporous Structure of Porous Carbon Monoliths Prepared through the Solvothermal Route

**DOI:** 10.3390/nano8040255

**Published:** 2018-04-19

**Authors:** Zhen Zhang, Junzong Feng, Yonggang Jiang, Ping Liu, Qiuhua Zhang, Ronghui Wei, Xiang Chen, Jian Feng

**Affiliations:** 1 Science and Technology on Advanced Ceramic Fibers and Composites Laboratory, National University of Defense Technology, 109 De Ya Rd, Changsha 410073, China; zhangzhen12a@126.com (Z.Z.); yayasummer0529@163.com (Y.J.); 2 Guangdong Alison Hi-Tech Co., Ltd., Qingyuan 513042, China; lp3779@163.com (P.L.); qiuhua6697@163.com (Q.Z.); weironghui@126.com (R.W.); laoshucx327@gmail.com (X.C.)

**Keywords:** self-sacrificial salt templating, porous carbon monoliths, solvothermal method, nanoporous structure

## Abstract

The conventional sol-gel method for preparing porous carbons is tedious and high-cost to prepare porous carbons and the control over the nanoporous architecture by solvents and carbonization is restricted. A simple and novel self-sacrificial salt templating method was first presented to adjust the microporous structure of porous carbon monoliths synthesized via the solvothermal method. Apart from good monolithic appearance, the solvothermal route allowed for ambient drying because it made sure that the polymerization reaction was completed quickly and thoroughly. The intact and crack-free porous carbon monoliths were investigated by scanning electron microscopy (SEM), thermogravimetric differential scanning calorimetry (TG-DSC), Fourier transform infrared (FT-IR), energy dispersive spectroscopy (EDS), X-ray photoelectron spectroscopy (XPS), X-ray diffraction (XRD) and nitrogen sorption measurements. It was proven that the self-sacrificial salts NH_4_SCN had been removed during pyrolyzing and so, porous carbon monoliths could be directly obtained after carbonization without the need of washing removal of salts. Most importantly, the microporous specific surface area of the resultant porous carbon monoliths was dramatically increased up to 770 m^2^/g and the Brunauer–Emmett–Teller (BET) specific surface area was up to 1131 m^2^/g. That was because the salts NH_4_SCN as self-sacrificial templating helped to form more around 0.6 nm, 0.72 nm and 1.1 nm micropores. The self-sacrificial salt templating is also a suitable and feasible method for controlling the nanoporous structure of other porous materials.

## 1. Introduction

Porous carbon materials including activated carbons, porous carbons, carbon foams and hollow carbon spheres, and so forth, are attracting more and more interest due to their unique characteristics of a high specific surface area, low density and intrinsic electrical conductivity as well as physicochemistry stability [1,2,3,4]. Among all kinds of porous carbons, porous carbon monoliths with the advantages of high porosity, ordered nanoporous arrangement and easy handling are potential candidates in the fields of separation, electrodes, supercapacitors, hydrogen storage, catalysts and so on [5,6,7]. Conventionally, porous carbon monoliths are obtained through pyrolyzing phenolic organic porous monoliths prepared through the sol-gel route and the supercritical drying process. With regard to the need of industrial application to keep low costs, high efficiency and be friendly to the environment, the traditional sol-gel methodology is not suitable for continuous production on a large scale because of tedious processes, high costs and hazardous operation as well as the use of pollutant organic solvents at the stage of supercritical drying [8,9,10,11,12,13].

Recently, the solvothermal method was used as a feasible, economical and ecofriendly technology to prepare porous carbon monoliths [14,15]. Through the solvothermal process, the phenolic sol-gel reaction developed quickly and thoroughly under a 100–200 °C temperature and high pressure resulting from the solvents themselves. Hence, the resultant organic wet gel possesses relatively high strength to provide the capillary force when ambient drying. The solvothermal pathway is becoming an important alternative for the preparation of porous carbon monoliths [16,17]. However, the solvothermal method has a limited control over the nanoporous structure of porous carbon monoliths. That is because mesopores and macropores can just be adjusted by solvents and the amount of micropores are just dependent on the carbonization temperature and duration. Although the activation method is usually applied to increase the amount of microporous architecture through postprocessing porous carbons by physical or chemical reaction, most of the activating agents, such as KOH, are toxic. Besides, the activation routine is always time-consuming and would decrease the yield of porous carbons [18,19,20,21,22]. Supramolecular templating is also a very common method for controlling the pores on the nanometer scale but this method is usually used to adjust the amount of mesopores or macropores and generally includes a post treatment to remove the supermolecular templating [23,24,25].

As a novel and feasible method, salt templating is invented to prepare carbon xerogel mostly for the application of energy storage, separation and so forth. Alkaline-earth metal salts are usually used as porogens and templates in the salt templating method [26]. Because the common salt templates are difficult to volatile or decompose even under high temperature during pyrolyzing, it is necessary for the pyrolyzed carbon xerogels to be washed several times and dried to remove salts. However, the step of washing for the removal of salts cannot make sure that salt templates are totally wiped off and at the same time, it is so time-consuming to delay the whole process cycle of the salt templating method. Recently, the use of self-sacrifice salt templating has been successfully used in sol-gel science not just to enhance the porosity but also to promote the formation of size- and shape controlled porosity [27,28,29].

In this work, a novel and simple method, self-sacrificial salt templating, was first put forward to control the nanoporous architecture of porous carbon monoliths prepared through the solvothermal route. Solvothermal treatment shortened the sol-gel aging time and, most importantly, endowed the organic porous framework with high strength to allow for ambient drying. Ammonium thiocyanates (NH_4_SCN) were selected to be self-sacrificial salt templates because their solubility was high and they could decompose at 190 °C. Through measurements including thermogravimetric differential scanning calorimetry (TG-DSC), Fourier transform infrared (FT-IR), energy dispersive spectroscopy (EDS), X-ray photoelectron spectroscopy (XPS) and X-ray diffraction (XRD), it was proved that salt templates had been removed in the process of carbonization. Hence, the porous carbon monoliths could be obtained after pyrolyzing without the need for washing removal. What is more, with the help of self-sacrificial salt templating, the number of micropores was dramatically increased, which was characterized through nitrogen sorption analyses. Self-sacrificial salt templating also provided a new and feasible choice for the synthesis of other nanoporous materials.

## 2. Materials and Methods

### 2.1. Material

Ammonium thiocyanate (NH_4_SCN) for self-sacrificial salt templating and methanol (CH_3_OH) for the solvent were purchased from Sinopharm Chemical Reagent Co., Ltd. (Shanghai, China) with purity at the analytic grade. The carbon precursor, including resorcinol (C_6_H_6_O_2_) and furfural (C_5_H_4_O_2_) and the catalyst hexamethylenetetramine (HMTA, C_6_H_12_N_4_), were also obtained from Sinopharm Chemical Reagent Co., Ltd. with a purity of >98%. All reagents were used as received without further purification.

### 2.2. Preparation of Porous Porous Carbon Monoliths

**Figure 1:**
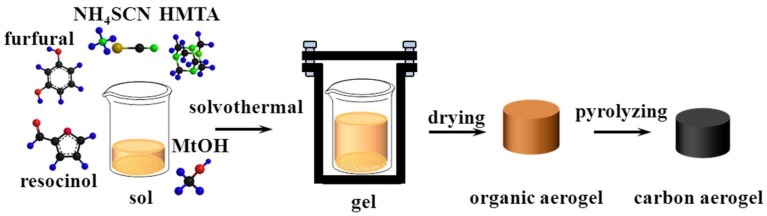
The schematic illustration of preparation of porous carbon monoliths.

As shown in Figure 1, the preparation processes of porous carbon monoliths could be divided into three main stages, that is, solvothermal gelling, drying and pyrolyzing. In a typical preparation of porous carbon monoliths, 3.69 g resorcinol and 9.61 g furfural as organic monomers and 2.54 g NH_4_SCN as salt templating and 0.04 g HMTA as catalysts were mixed and stirred thoroughly to obtain black-brown clear liquids. And then, 26.98 mL of methanol was dropwise added and stirred for 1 h to yield a transparent sol. After that, the sol was sealed in a 100 mL Teflon-lined autoclave (with an additional cylinder glass container for achieving a good monolithic appearance) and went on solvothermal treatment at 150 °C for 15 h. Subsequently, the resultant wet gel was dried to acquire an organic porous monolith in a vacuum oven at 100 °C overnight. Finally, the porous carbon monolith was prepared through pyrolyzing the porous organic polymer under a flowing argon atmosphere at 900 °C for 1 h (heating rate: 2 °C/min). For comparison, the other samples were synthesized through a similar approach, except replacing the amount of NH_4_SCN with 0, 1.26 and 3.81 g, respectively. The resulting porous carbon monoliths were named N/R-t where N represented NH_4_SCN, R was resorcinol and t was determined by the amount of NH_4_SCN.

### 2.3. Characterization of Porous Carbon Monoliths

Thermogravimetric and differential scanning calorimeter analyses (TG-DSC) of porous carbon monoliths and porous organic monolith were obtained through a NETZSCH STA449F3 thermal analyzer (Selb, Germany). The scanning electron microscopy (SEM) images and energy dispersive X-ray data were collected using a CMSS4800 instrument (Tokyo, Japan). The X-ray diffraction patterns were acquired recorded by a D8 Advance X-ray diffractometer (Braunschweig, Germany). X-ray photoelectron spectra of porous carbon monoliths were determined using an ESCALAB 250Xi equipment (Waltham, MA, USA). Fourier transform infrared spectroscopy was measured on a Nicolet avatar 360 spectrometer (Waltham, MA, USA). The nanoporous structure was characterized through a 3H-2000PM2 apparatus from BeiShiDe Instrument (Beijing, China). The Brunauer–Emmett–Teller (BET) specific surface area was acquired using the desorption isotherm and the pore size distribution curve was calculated from the adsorption branch applying the slit pore non-local density functional theory. The microporous volume and microporous surface area were determined by the t-plot method. The external surface area is the BET specific surface area minus the micropore specific surface area. The mass density was directly obtained through the mass of sample divided by its volume.

## 3. Results

### 3.1. Proofs of Self-Sacrificial Salt Templating

**Figure 2:**
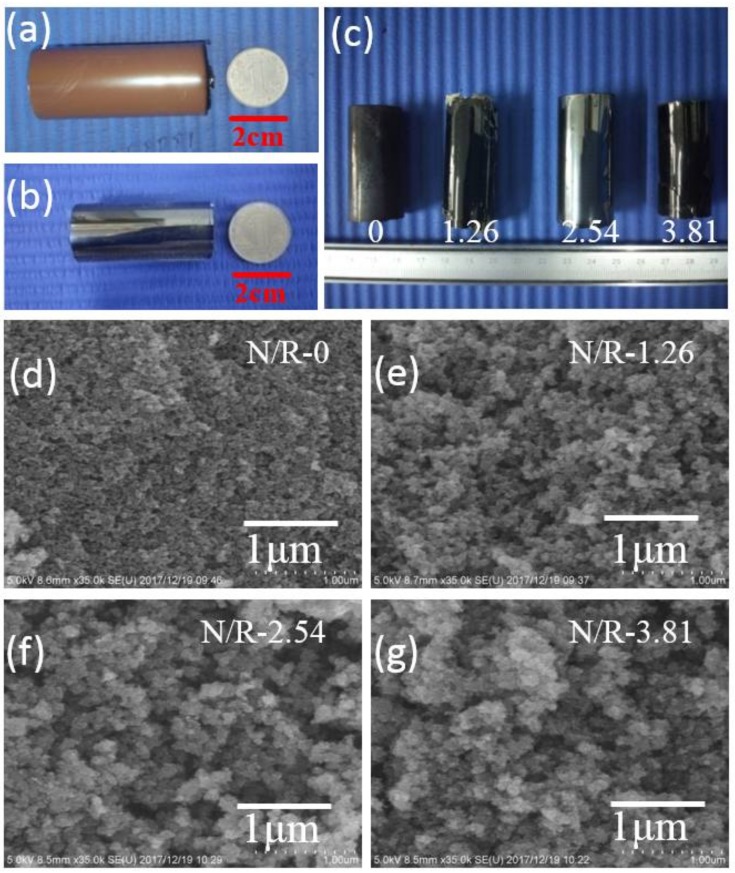
Photographs of the porous organic monolith (**a**), porous carbon monolith for N/R-2.54 (**b**) and four porous carbon monoliths for N/R-0, N/R-1.26, N/R-2.54 and N/R-3.81 in turn (**c**). Scanning electron microscopy (SEM) micrographs of the porous carbon monoliths N/R-0 (**d**), N/R-1.26 (**e**), N/R-2.54 (**f**) and N/R-3.81 (**g**).

The photographs of porous organic monolith and four porous carbon monoliths are exhibited in Figure 2a–c. As could be seen from Figure 2a, an intact and crack-free brown cylinder was obtained after solvothermal gelation and ambient drying. Apart from shortening the gelling and aging time and solvothermal treatment endowed the organic gel with a robust organic skeleton and high strength by promoting and accelerating condensation polymerization between resorcinol and furfural. Moreover, methanol with low surface tension was chosen to be the solvent to preserve the nanostructure during ambient drying. Two crucial reasons made sure that a whole and flawless porous organic monolith could be prepared after ambient drying: No crystal salts existed on the surface of the porous organic monolith and this reflected that there was no obvious phase separation during the polymerization stage. From the digital photographs of porous carbon monoliths in Figure 2b,c, the synthesized porous carbon monoliths were even, smooth and without cracks; last but not least, there were no crystal substances from salts on the surface of the porous carbon monoliths, which preliminarily reflected that NH_4_SCN salts had already pyrolyzed or volatilized in the process of carbonization [30].

Scanning electron microscopy (SEM) images depicted in Figure 2d–g show that the porous carbon monoliths were composed of an interconnected nanoporous network skeleton architecture with a diameter of roughly 10–50 nm and the typical porous structure was homogeneous and uniform, which reflected that the obtained porous carbons possessed some typical characters of carbon aerogels. From the SEM photographs, only the mesopores and macropores can be observed. When the amount of NH_4_SCN increased from 0 g to 1.26 g to 2.54 g, the concentration of mesopores and macropores gradually increased. However, from N/R-2.54 to N/R-3.81, the porous structure seemed to reduce and the robust skeleton tended to be loose with some macropores collapsing. The results were caused because the salt NH_4_SCN played the role of templating agent. With the concentration of NH_4_SCN increasing, the salt templates occupied more volumes in the second particles and then helped to form a more porous structure as porogens. However, the excess salt template could lead to generating too many pores and so the weak architecture might collapse and be destroyed, which is discussed in detail below [31].

**Figure 3:**
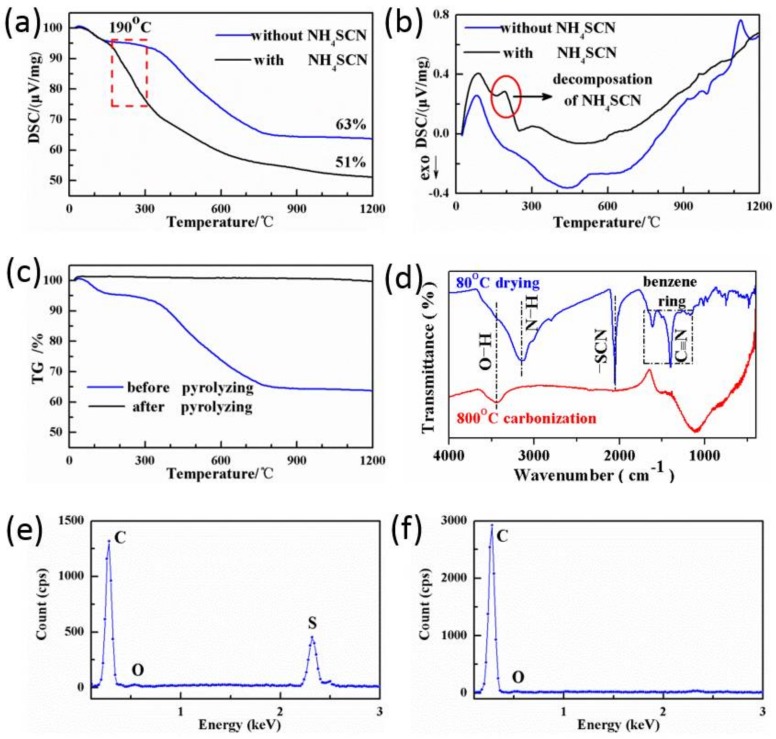
(**a**) The thermogravimetric (TG) traces of porous organic monolith for N/R-0 and N/R-2.54, (**b**) the differential scanning calorimetry (DSC) traces of porous organic for N/R-0 and N/R-2.54, (**c**) the TG traces of N/R-2.54 samples before and after pyrolyzing, (**d**) the Fourier transform infrared (FT-IR) patterns of N/R-2.54 samples before and after pyrolyzing, (**e**,**f**) the energy dispersive spectroscopy (EDS) curves of N/R-2.54 samples before and after pyrolyzing, respectively.

Figure 3a shows the thermogravimetric (TG) traces of porous organic monolith for N/R-0 and N/R-2.54. The mass loss of N/R-2.54 between 180 and 250 °C was more than that of N/R-0 and there was no doubt that the reason should be attributed to NH_4_SCN salts. Furthermore, the differential scanning calorimetry (DSC) traces of the two samples in Figure 3b also indicated that a particular endothermic peak appeared at around 190 °C for N/R-2.54 porous organic polymer. The differences were created by the thermal decomposition of NH_4_SCN at 190 °C. The important information illustrated that NH_4_SCN salts had already started to self-decompose at low temperatures in the process of carbonization. From Figure 3c, notably, the obtained porous carbons had little mass loss even at 1200 °C and this proved that upon pyrolyzing, the porous carbons possessed very great thermal stability under inert atmosphere. As for the FT-IR patterns of N/R-2.54 sample before and after pyrolyzing in Figure 3d, the characteristic band ascribed to –SCN at about 2050 cm^−1^ vanished after pyrolyzing, which suggested that no NH_4_SCN salts existed throughout carbonization [32,33]. Figure 3e,f show the EDS analyses. The EDS results demonstrated that the content of S element from NH_4_SCN was negligible and this further showed that the NH_4_SCN salts had been removed after carbonization. The EDS data of N/R-2.54 samples before and after pyrolyzing are listed in Table 1. Because the N element cannot be measured with accuracy, the EDS analysis just included C, O and S elements. After pyrolyzing, the amount of S, coming from NH_4_SCN, decreased to 0.55%. Hence, the porous carbon monoliths can be directly prepared through pyrolyzing with no need for tedious washing with water.

**Figure 4:**
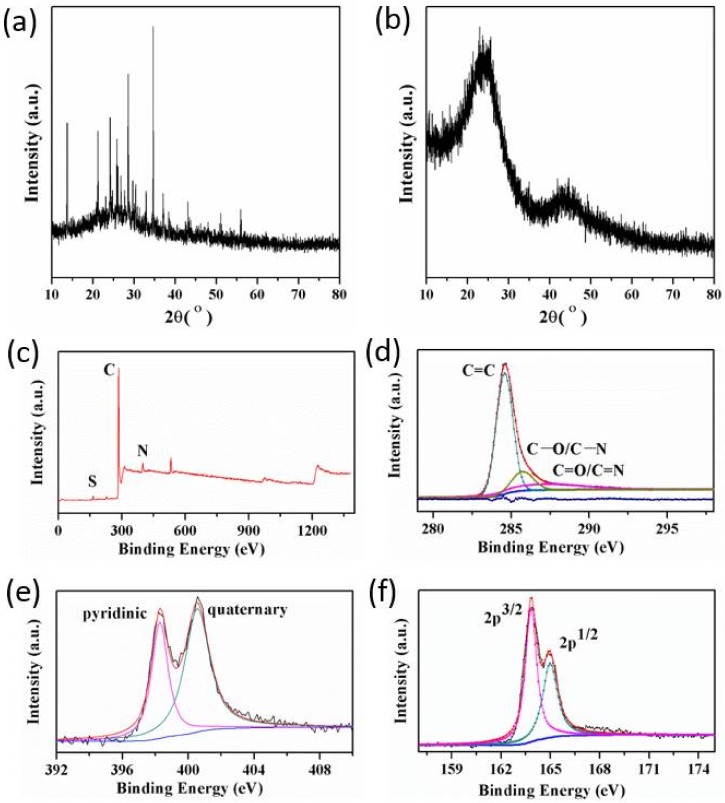
The X-ray diffraction (XRD) spectra of N/R-2.54 samples before (**a**) and after (**b**) pyrolyzing. The X-ray photoelectron spectroscopy (XPS) survey spectrum (**c**), high resolution XPS fine spectrum of C1s orbit region (**d**), N1s orbit region (**e**) and S2p orbit region (**f**) of porous carbon for N/R-2.54.

The proof of self-sacrificial salt templates is further confirmed by XRD patterns. The wide-angle XRD patterns of N/R-2.54 samples before (a) and after (b) pyrolyzing are exhibited in Figure 4a,b. Prior to pyrolyzing, the pattern for the N/R-2.54 porous organic monolith had a number of peaks that could be well-indexed to NH_4_SCN while after pyrolyzing, no characteristic diffraction peaks of NH_4_SCN but only two broad features resulted from amorphous porous carbons were observed. And this implied that NH_4_SCN had been decomposed after pyrolyzing, which was consistent with the above analyses from TG-DSC and FT-IR. XPS was used to study the elements on the surface and to characterize the C and N as well as the S state from NH_4_SCN. The XPS data of N/R-2.54 samples before and after pyrolyzing were listed in Table 2. Through comparing the amount of S element, the XPS results were basically consistent with the EDS results. The amount of S element is very low and can be ignored. So, we can conclude that the NH_4_SCN has been removed after pyrolyzing at a high temperature. The remnant S resulted from a little NH_4_SCN reacting with carbon matrix to link to the carbon skeleton before decomposing at a high temperature. In addition, the XPS spectra in Figure 4c–f are used to study the surface species and investigate the atom binding states of N/R-2.54 porous carbon. From Figure 4c, there was the additional presence of N and S elements and there was no doubt that they came from NH_4_SCN. In the case of the C1s spectrum in Figure 4d, the peaks at around 284.6 eV, 286.3 eV and 288.5 eV contributed to sp2 hybridized C, single C bonded to S, O or N and double C bonded to S, O or N, respectively. According to the N1s fine spectrum in Figure 4e, the N/R-2.54 porous carbon contained pyridinic (at 398.5 eV) and quaternary (at 400.9 eV) nitrogen species. Interestingly, the relative amount of quaternary nitrogen was more than that of pyridinic nitrogen. Because the initial N state from NH_4_SCN prior to annealing was –C≡N, the presence of both pyridinic and graphitic nitrogen indicated that some of the thiocyanide had crosslinked with organic phenolic when pyrolyzing. Similarly, the S species in Figure 4f exhibited C-bound S2p orbit doublet C–S–C 2p3/2 and 2p1/2 at around 163.6 eV and 165.1 eV, respectively. These results confirmed that, before decomposing, a little of the NH_4_SCN crosslinked with the carbon matrix at the high temperature during carbonization [34,35,36]. With regard to the little dopant of N and S, the S and N doped porous carbon monoliths may have great application prospects in the fields of catalysts, electrodes and supercapacitors.

### 3.2. Control over the Nanoporous Architecture of Porous Carbon Monoliths

**Figure 5:**
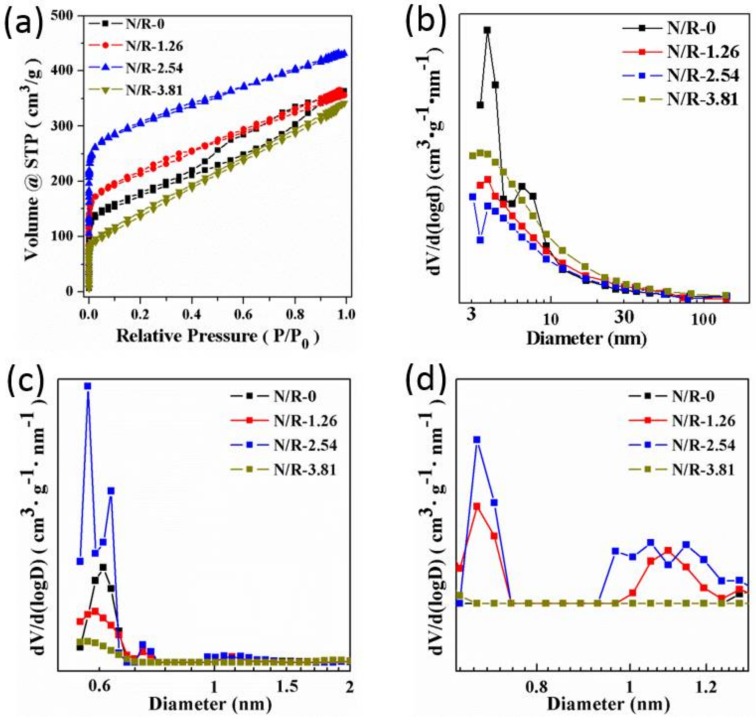
The nitrogen sorption isotherms (**a**), BJH pore size distribution curves (**b**) and DFT pore size distribution curves (**c**,**d**) of porous carbons for N/R-0, N/R-1.26, N/R-2.54 and N/R-3.81.

The nitrogen sorption isotherms and pore size distribution curves in Figure 5 are used to analyze the nanoporous structure of porous carbon monoliths. From the nitrogen sorption of porous carbons for N/R-0, N/R-1.26, N/R-2.54 and N/R-3.81, it could be seen that all of the isotherms were intermediate between type-I and type-IV according to the IUPAC classification (International Union of Pure and Applied Chemistry), which exhibited the typical features of microporous and mesoporous materials, respectively. BJH (Brunauer–Emmett–Teller) pore size distribution curves calculated from the desorption branches of isotherms in Figure 5b also confirmed that they are mesoporous. Most importantly, as the concentration of NH_4_SCN salts was enhanced, the adsorption amount in the low relative pressure P/P_0_ region between 0 and 0.1 initially increased but subsequently decreased. To further study the changes of microporous structure, the DFT (Density Functional Theory) pore size distribution curves in Figure 5c,d are determined from the slit pore NLDFT (Nonlocal Density Functional Theory) model. From the whole micropore size distribution plot in Figure 5c and magnified micropore size distribution plot in Figure 5d, N/R-2.54 samples had the most relative distribution of micropores. This difference should be attributed to NH_4_SCN salt templates. The salt templating as ionic pairs and small clusters formed more micropores.

The textural properties of porous carbons for N/R-0, N/R-1.26, N/R-2.54 and N/R-3.81 are provided in Table 3. It was clear that the density of the porous carbon monoliths was as low as 0.13 cm^3^/g and the increase of added NH_4_SCN salt templates could lead to the decrease of porous carbon density through the formation of more pore architecture. The initial mesopores of the N/R-0 sample are formed due to the solvents, which have been proved many times in the literature. The solvents play the role of dispersant and create voids between carbon particles. After drying, the spaces occupied by solvents become the pore structure. With the addition of NH4SCN, the pore size increases. If the increase in the pore size was because of phase separation, the increased pores should be mesopores and macropores. However, in fact, it is the micropores, instead of the mesopores, that increased. Hence, the larger pores should be attributed to the formation of more micropores, which linked to one another to form mesopores. The BET specific surface area, micropore specific surface area, micropore volume, as well as pore volume, were the largest when the amount of NH_4_SCN was 2.54 g. The content of mesopores rose with the added NH_4_SCN salts increasing. According to the shift trend of micropores, there was an optimal additive amount of NH_4_SCN to enhance the micropore specific surface area and total pore volume. As the added amount of NH_4_SCN increased, the micropores continuously increased and the BET specific surface area was successively enhanced. When the amount of NH_4_SCN was excessive, too many micropores linked to form mesopores with the decrease of the BET specific surface area and the superfluous and interconnected mesopores might not afford the surface tension to collapse during the stage of ambient drying, which corresponded to the observation from the SEM photographs.

## 4. Conclusions

Through solvothermal method-assisted with self-sacrificial salt templating, the intact and crack-free porous carbon monoliths with a BET specific surface area up to 1131 m^2^/g were synthesized. It had been proven that the selected salts NH_4_SCN as self-sacrificial templates could decompose after carbonization. Therefore, porous carbon monoliths could be directly obtained after pyrolyzing. Self-sacrificial salt templates played the role of enhancing the number of micropores and so, the microporous specific surface area of the as-prepared porous carbon monoliths was increased to 770 m^2^/g due to forming more 0.6 nm, 0.72 nm and 1.1 nm micropores. Due to high microporosity and monolithic advantages, the porous carbon monoliths have great application potential in many fields such as ultra-high thermal insulation, acoustic insulation and so on. The self-sacrificial salt templating offers a new way to adjust the nanoporous structure of porous materials.

## Figures and Tables

**Table 1:** EDS data of N/R-2.54 samples before and after pyrolyzing.

Sample	Element Weight (%)		
	C	O	S
N/R-2.54 before pyrolyzing	87.60	2.98	9.42
N/R-2.54 after pyrolyzing	93.35	6.10	0.55

**Table 2:** XPS data of N/R-2.54 samples before and after pyrolyzing.

Sample	Element Weight (%)		
	C	N	S
N/R-2.54 before pyrolyzing	74.40	15.87	9.73
N/R-2.54 after pyrolyzing	97.54	1.51	0.95

**Table 3:** The textural properties of porous carbons for N/R-0, N/R-1.26, N/R-2.54 and N/R-3.81.

Samples	Density (g/cm^3^)	BET Specific Surface Area (m^2^/g)	Pore Volume (cm^3^/g)	Micropore Volume (cm^3^/g)	Micropore Specific Surface Area (m^2^/g)	External Surface Area (m^2^/g)
N/R-0	0.21	611	0.562	0.099	213	398
N/R-1.26	0.18	761	0.551	0.142	326	435
N/R-2.54	0.16	1131	0.667	0.316	770	361
N/R-3.81	0.13	532	0.531	0.105	125	407
